# Characterization of the complete chloroplast genome of a hybrid species *Cymbidium tracyanum var ‘huanghua’* × *Cymbidium faberi cv ‘huanghui’*

**DOI:** 10.1080/23802359.2020.1848467

**Published:** 2021-04-23

**Authors:** Yiran Zhao, Zhilin Li, Longjie Cheng, Yefang Li, Fengmei He, Yuying Wang

**Affiliations:** College of Horticulture and Landscape, Yunnan Agricultural University, Kunming, China

**Keywords:** *Cymbidium tracyanum var ‘huanghua’×Cymbidium faberi cv ‘huanghui’*, chloroplast genome, Endangered species, phylogenetic analysis

## Abstract

*Cymbidium tracyanumvar* ‘*huanghua*’ × *Cymbidium fabericv* ‘*huanghui*’ is a hybrid species with important ornamental *value.* In the research, Illumina high-throughput sequencing technologies was used to sequence the chloroplastic genome of *Cymbidium tracyanumvar* ‘*huanghua*’ × *C. fabericv* ‘*huanghui*’. The genome features of *Cymbidium tracyanumvar* ‘*huanghua*’ × *C. fabericv* ‘*huanghui*’ and the phylogenetic relationships were reported and established. The total length of the complete chloroplast genome is 154,741 bp, consisting of a pair of inverse duplication regions 26,968 bp, a large single-copy region 84,410 bp and a small single-copy region 16,395 bp. The complete genome contains 73 protein-coding genes (PCGs), The entire genome contains 73 genes that encode proteins, 30 tRNA genes and 4 rRNA genes. The phylogenetic tree revealed that *Cymbidium tracyanumvar* ‘*huanghua*’ × *C. fabericv* ‘*huanghui*’ is more closely related to *Cymbidium erythraeum.*

*Cymbidium tracyanumvar* ‘*huanghua*’× *C. fabericv* ‘*huanghui*’ is a hybrid species. It is a cross between wild resources of *Cymbidium tracyanumvar* ‘*huanghua*’ Baoshan in the southwest of Yunnan Province and wild resources of *C. faberi* Dali in the west of central Yunnan Province. It was bred by the College of Horticulture and Landscape, Yunnan Agricultural University in 2010. Both of *Cymbidium tracyanum* and *C. faberi* hybrid parents belong to a ClassIprotected plant in the China Rare and Endangered Plants List (http://www.iplant.cn/rep/protlist). The*Cymbidium tracyanumvar* ‘*huanghua*’× *C. fabericv* ‘*huanghui*’ is an excellent ornamental garden plant with yellow flowers, unique scent, beautiful flower shape.The length of inflorescence is 30–80 cm with 10–20 or more flowers, and the flowering period ranges from October to January of the following year (Liu et al. 2009). Therefore, it is suitable for potted or cut flowers.

The complete chloroplast genome sequences of *Cymbidium tracyanumvar* ‘*huanghua*’ × *C. fabericv* ‘*huanghui*’was obtained (GenBank Accession No.MT675524). The genome sequences and features can be used to study the phylogenetic relationship of *Cymbidium tracyanumvar* ‘*huanghua*’× *C. fabericv* ‘*huanghui*’ and contribute to the further research of chloroplast. In addition, it plays an essential role in the diversity research of genetic resources of this plant.Specimens were gathered from Yunnan province (China; 25°07′43″N, 102°44′54″E), and specimens were deposited in the Herbarium of Kunming Institute of Botany of CAS (specimen code: CY004). Specimens were deposited in the College of Horticulture and Landscape, Yunnan Agricultural University. The total chloroplast DNA of *Cymbidium tracyanumvar* ‘*huanghua*’ × *C. fabericv* ‘*huanghui*’ was extracted from fresh leaf mesophyll tissue. The method of DNA extraction is CTAB (Doyle and Doyle 1987).

Sequencing the DNA was performed using the Illumina NovaSeq in GENOSEQ Technologies Limited Company (Wuhan, China). Namely, the raw data were obtained and then were assembled by SPAdes (Dierckxsens et al. 2016). The assembled contigs were compared with the chloroplast genomes of the closely related species through the use of blastn (version: BLAST 2.2.30+, parameter: -evalue 1e-5). Then the contigs were checked, selected, adjusted to get the final data. The chloroplast genome was annotated and mapped by using GeSeq (Tillich et al. 2017). A total of 24 complete chloroplast genomes of *Orchid* species were used to investigate the phylogenetic position of *Cymbidium tracyanumvar* ‘*huanghua*’ × *C. fabericv* ‘*huanghui*’.

The length of complete chloroplast genome of *Cymbidium tracyanumvar* ‘*huanghua*’ × *C. fabericv* ‘*huanghui*’ is 154,741 bp. The genome presented a characteristic quadripartite circular structure which included one pair of inverted repeat regions (IRs, 26,968 bp), one large single-copy region (LSC, 84,410 bp) and one small single-copy region (SSC, 16,395 bp). Besides, the complete genome contains 73 messenger RNA genes, 30 transfer RNA genes and 4 ribosomal RNA genes. The overall GC content of *Cymbidium tracyanumvar* ‘*huanghua*’× *C. fabericv* ‘*huanghui*’ chloroplast genome is 36.83%. Moreover, the GC content of IR regions (42.94%) is higher than the LSC region (34.32%) and the SSC region (29.60%).

To study the phylogenetic relationship of *Cymbidium tracyanumvar* ‘*huanghua*’ × *C. fabericv* ‘*huanghui*’, a phylogenetic tree was constructed by using 19 complete chloroplast genomes of *Cymbidium*. Using *Paphiopedilum armeniacum* (LC085347)*, Eria corneri* (MN477202) and *Dendrobium thyrsiflorum* (NC047439) as the outgroups. All the sequences were downloaded from NCBI GenBank. All sequences of species were aligned by the online program MAFFT version 7.0 and MEGA version 7.0 was used to build the maximum-likelihood phylogenetic tree with 1000 rapid bootstrap replicates (Kumar et al. 2016). The phylogenetic analysis result showed that *Cymbidium tracyanumvar* ‘*huanghua*’ × *C. fabericv* ‘*huanghui*’ was closely related to *Cymbidium erythraeum* (Figure 1).

**Figure 1.**
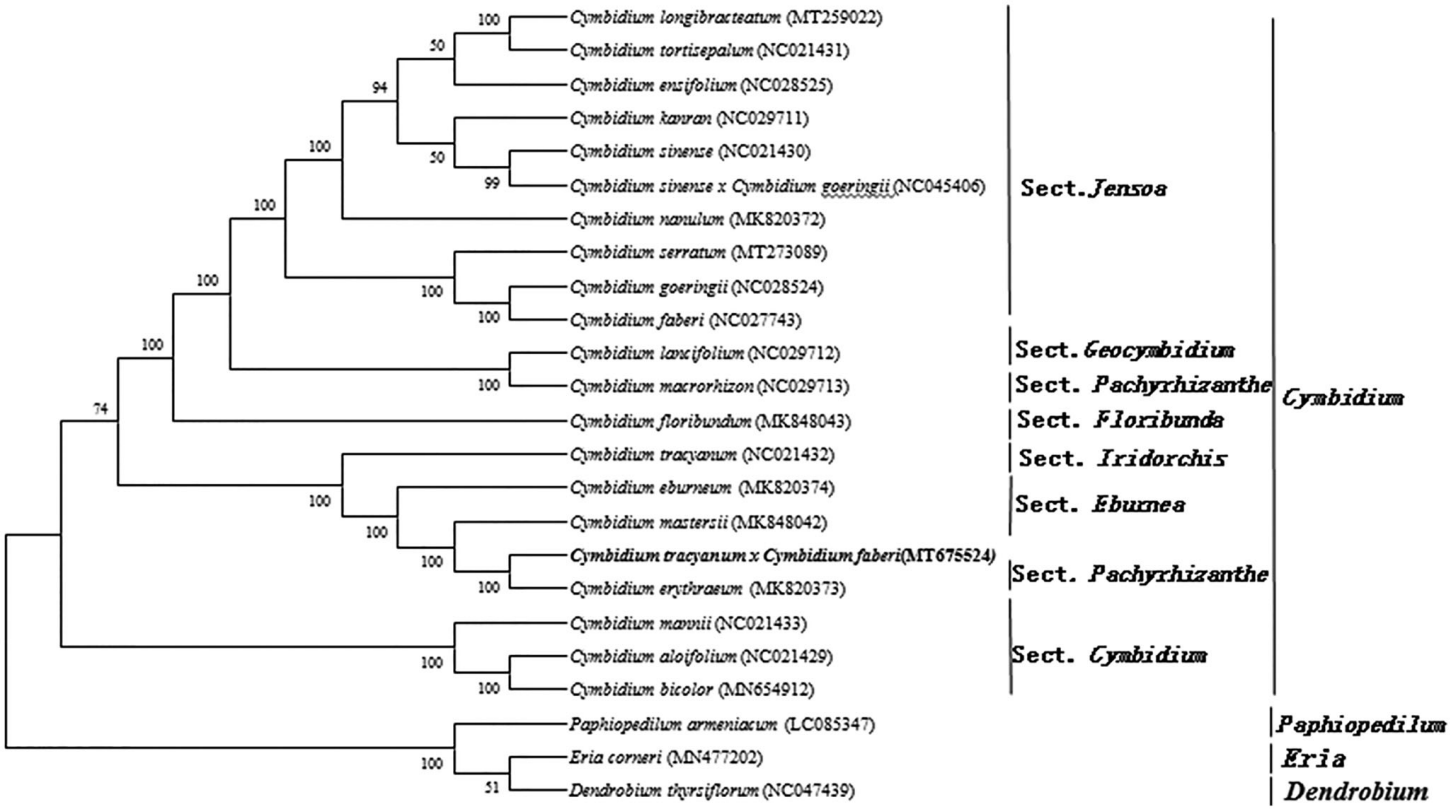
A phylogenetic tree based on 24 complete chloroplast genome sequences of *Orchidaceae* species using the maximum likelihood (ML) analysis by MEGA version 7.0. Bootstrap support values are indicated in each node.

## Data Availability

The data that support the findings of this study are openly available in GenBank of NCBI at https://www.ncbi.nlm.nih.gov, reference number MT675524.
